# The Availability, Cost, Limitations, Learning Curve and Future of Robotic Systems in Urology and Prostate Cancer Surgery

**DOI:** 10.3390/jcm12062268

**Published:** 2023-03-15

**Authors:** Thomas Hughes, Bhavan Rai, Sanjeev Madaan, Edmund Chedgy, Bhaskar Somani

**Affiliations:** 1 Core Surgical Trainee, Warwick Hospital, Warwick CV34 5BW, UK; 2 Consultant Urologist and Robotic Surgeon, Freeman Hospital, Newcastle NE7 7DN, UK; 3 Consultant Urological Surgeon, Darent Valley Hospital, Dartford & Gravesham NHS Trust, Dartford DA2 8DA, UK; 4 Consultant Urological Surgeon, University Hospital Southampton, Southampton SO16 6YD, UK

**Keywords:** robotic surgery, learning curves, prostatectomy, robotic systems

## Abstract

Robot-assisted surgical systems (RASS) have revolutionised the management of many urological conditions over the last two decades with robot-assisted radical prostatectomy (RARP) now being considered by many to be the preferred surgical approach. Intuitive Surgical has dominated the market during this time period with successive iterations of the da Vinci model. The expiration of patents has opened the RASS market and several new contenders have become available or are currently in development. This comprehensive narrative review aims to explore the merits of each robotic system as well as the evidence and barriers to their use. The newly developed RASS have increased the versality of robotic surgical systems to a wider range of settings through advancement in technology. The increased competition may result in an overall reduction in cost, broadening the accessibility of RASS. Learning curves and training remain a barrier to their use, but the situation appears to be improving through dedicated training programmes. Outcomes for RARP have been well investigated and tend to support improved early functional outcomes. Overall, the rapid developments in the field of robot-assisted surgery indicate the beginning of a promising new era to further enhance urological surgery.

## 1. Introduction 

The advent of robot-assisted surgery (RAS) in the early 2000s has revolutionised the management of many urological conditions, particularly prostate cancer, with robot-assisted laparoscopic radical prostatectomy (RARP) now being considered by many to be the preferred surgical approach [1,2]. By 2017–2019, 88% of all radical prostatectomies in the UK were robot-assisted, a significant growth since the first ever RARP was performed in Germany in 2000 [3,4]. RAS has the benefits of minimally invasive surgery compared to open surgery with faster recovery, shorter length of stay and reduced tissue trauma, whilst also overcoming many of the challenges faced by laparoscopy [1,5,6]. RAS provides a 3D, magnified visualisation of the surgical field and improved ergonomics for the surgeon with instruments allowing greater dexterity [7]. Since its introduction, there has been a rapid uptake and adoption of RAS in many branches of medicine and surgery [8,9,10,11]. 

Within the field of urology, RAS systems have been utilised in a wide variety of oncological and non-oncological conditions, including cystectomy with intracorporeal or extracorporeal urinary diversion, partial or radical nephrectomy, retroperitoneal lymph node dissection (RPLND), adrenalectomy, pyeloplasty and artificial urinary sphincter insertion [10,11]. Robot-assisted radical cystectomy (RARC) has been associated with increased operative time but also a reduced length of stay compared to an open approach [12]. Robot-assisted partial nephrectomy has been associated with a reduced rate of conversion to open surgery compared to a laparoscopic approach, a reduced length of stay and a smaller reduction in glomerular filtration rate [13]. Robotic RPLND has been associated with reduced overall complication rate and reduced blood transfusion compared to open RPLND, but there was a 9% rate of conversion to open surgery in post-chemotherapy patients [14].

Since first approval by the US Food and Drug Administration (FDA) in 2000 for human use, the da Vinci surgical system (Intuitive Surgical, Sunnyvale, CA, USA) has dominated the market for more than 20 years to the extent that da Vinci has become synonymous with robotic surgery. The market dominance achieved through early successes and judicious patenting of technological developments appears to be coming to an end [8]. The lack of competition has allowed Intuitive Surgical to monopolise the global surgical robotics market, which was estimated at USD 3.6 billion in 2021 with a predicted annual growth rate of 19.3% from 2022 to 2030 [15]. Intuitive Surgical reported that almost 1.6 million operations were performed with the da Vinci systems in 2021 alone [16]. In the last few years, several alternative robotic surgical systems have been announced and are at various stages of development and commercial availability [17,18,19,20]. This heralds an exciting time for robotic surgery as the increased competition will inevitably drive forward technological advancements and cost reduction. 

However, the current costs associated with RAS can be prohibitively expensive to many regions, limiting the use of robotic surgery [21]. Another challenge is the need to adequately train surgeons to competently perform RAS, with each procedure coming with its own learning curve [22]. Furthermore, mechanical failure of robotic surgery systems can rarely result in patient harm or the need to convert to an open procedure.

This comprehensive review article proposes to review the current availability of robotic systems in urology with a focus on prostate cancer surgery, regarding the evidence to support their use and limitations of RAS, including the associated costs and barriers to their adoption. 

## 2. History of Robotics in Urology

Robotics was first introduced into urology in the 1980s when the PROBOT was developed as an autonomous robot to perform robotic transurethral resection of the prostate [23]. Although these trials proved to be safe and successful, the PROBOT was never widely produced and implemented. In the latter half of the 1990s, there was increasing interest in ‘master-slave’ robotic technology with a view to improving laparoscopic surgery. ZEUS (Computer Motion, Goleta, CA, USA) and da Vinci (Intuitive Surgical, Sunnyvale, CA, USA) were developed at a similar time; however, the seven degrees of freedom (DoF) afforded by the four arms of the da Vinci system with its 3D binocular imagery proved superior [24]. A merger between Intuitive and Computer Motion in 2003 paved the way for 20 years of dominance by successive generations of Intuitive’s da Vinci systems. 

## 3. Robotic Systems

Robot-assisted surgical systems (RASS) are designed to overcome many of the shortcomings of conventional laparoscopic surgery. Whilst laparoscopic surgery undoubtedly has benefits in reducing tissue trauma and reducing the length of hospital stay for patients compared to open surgery, it does have some negative aspects [7]. Most are related to the ergonomics of laparoscopic surgery, where there is a high burden on surgeons and their assistants to hold equipment, retract tissue and manipulate the camera, which can be associated with negative health impacts, including shoulder and hand pain, due to unnatural working angles [25]. Robots take on this role, freeing clinicians and assistants to perform alternative tasks. Robotic surgery facilitates greater discriminatory movements afforded by seven DoF compared to four DoF for conventional laparoscopic surgery [7]. Alongside the greater dexterity and discriminatory movements offered by RAS, other benefits included tremor filtration and 3D visualisation that offers an improved field of view, particularly in the pelvis [1]. 

Developments in surgical technique have also been improved with the increased exposure and visibility afforded by RAS. Retzius-sparing RARP approaches the prostate from the posterior aspect of the bladder, which is a small space that would be inaccessible with laparoscopic surgery [9]. Reported benefits include improved early continence using a Retzius-sparing technique, but this difference has diminished at twelve months [9,26]. Although there have been some concerns regarding positive surgical margins when using a Retzius-sparing technique, a recent meta-analysis suggests these may be unfounded [26].

Longer term outcomes are also being reported for patients undergoing RAS. A multi-centre prospective study comparing RARP to open retropubic radical prostatectomy (RRP) found lower rates of erectile dysfunction, prostate cancer-specific mortality and biochemical recurrence for the RARP group after 8 years of follow-up, although despite 4000 participants, this study was limited by the lack of randomisation [27]. 

## 4. Da Vinci

**Figure 1:**
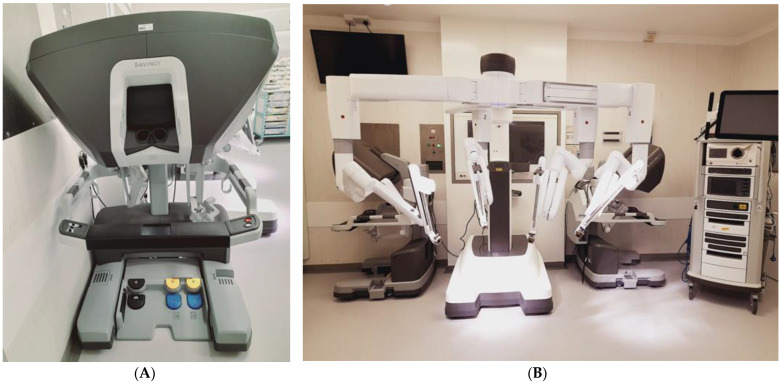
Da Vinci Xi Robotic System (**A**) Surgeon’s Console. (**B**) Robot cart with 4 arms deployed in centre of image, the vision cart is to the right of the image.

Intuitive Surgical currently offers both multiport and single-port RAS systems with the X/Xi and SP models currently available. The da Vinci X/Xi consists of a patient cart, surgeon console away from the operating table, and a vision cart [28] (Figure 1). The closed surgeon console provides a 3D high-definition view and the surgeon uses the hand controls from a seated position to manipulate the robotic instruments [28]. The multi-arm patient cart utilises 8 mm diameter instruments and an 8 mm camera, allowing versatility of camera placement and therefore facilitating multi-quadrant procedures [28]. The da Vinci Xi incorporates EndoWrist technology which simulates the movements of a human wrist with seven DoF and is coupled with a tremor-filtering system for smooth, controlled movements [28].

The da Vinci single-port (SP) system was approved for use in urological surgery in 2018 by the FDA. This system has a 25 mm multichannel port at the sole entry point, which incorporates a 12 × 10 mm articulating endoscopic camera and three double-jointed articulating instruments [29]. An additional assistant port is typically employed, usually in the right lower quadrant for RARP, for example [30,31]. As with the Xi, a dual surgeon console setup can facilitate training of surgeons [29]. A recent meta-analysis suggests equivalence of initial outcomes between single-port and multi-port RAS for RARP in terms of blood loss and operative time, with SP systems associated with having a shorter length of stay [32]. However, the studies included are predominately single-centre case series and due to the nascency of SP RASS, longer term functional and oncological outcomes are not yet known [32]. The technical differences between a SP and multi-port systems inevitably means that there will be a learning curve required to transition to single-port system, even for surgeons with substantial experience at performing RARPs using multi-port systems [30].

## 5. Versius 

**Figure 2:**
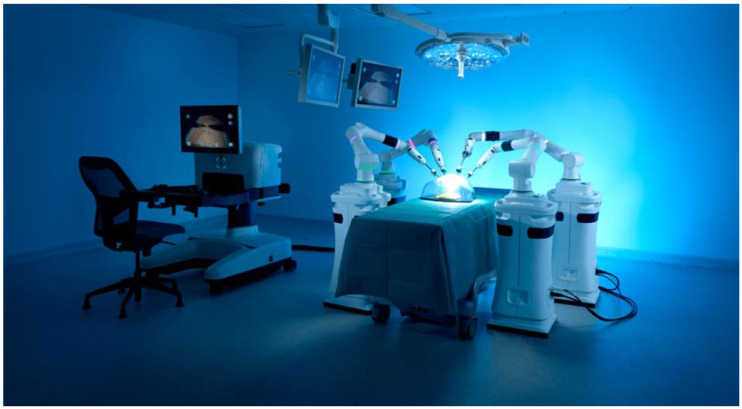
Versius Robotic System [33]. The open surgeon’s console configured in the seating position is shown on the left of the image. The four individual robotic arm units are arranged around the operating table. Image reproduced with permission from CMR Surgical (Cambridge, UK).

The Versius surgical system (CMR Ltd., Cambridge, UK) has been licensed for use in Europe as of March 2019 [33] (Figure 2). It features an open surgeon’s console that can be configured to allow for a sitting or standing position and is compact with individual bedside units for each robotic arm [18]. V-wrist technology is implemented, allowing seven DoF and 360 degrees of wrist motion to manipulate the sterilisable 5 mm instruments [18]. Whilst preclinical evaluation has been carried out with cadaveric nephrectomies and prostatectomies, there is a lack of clinical data published to date [34]. Early clinical experiences in colorectal surgery and gynaecology suggest that the Versius system is safe, but a greater body of evidence is required to substantiate these findings [35]. Nevertheless, CMR have reported that over 5000 clinical cases have now been performed across their 100 installed robots in November 2022 [36].

## 6. Senhance

The Senhance (Asensus Surgical, Durham, NC, USA) robotic system received FDA approval in 2017, but this was limited to general surgical and gynaecological procedures. Described as a digital laparoscopy system, with instrument controls at the surgeon console providing haptic feedback to the surgeon and resembling traditional laparoscopic instruments, it offers an easier transition to robotic surgery for those trained in laparoscopic surgery [17]. The console is open in design, allowing for greater teamwork, and the 3D imagery is obtained via specialist polarised glasses. The system also uses an eye-tracking control to manipulate the camera to adjust the field of view [17]. The four robotics arms have their own cart and the instruments utilised have diameters of 3, 5 and 10 mm and are reusable after sterilisation, reducing costs [17]. Although there are a few European case series from Lithuania and Croatia, the uptake has been limited in the US by a lack of FDA approval for urological surgery [37,38]. 

## 7. Hinotori

The Hinotori (Medicaroid Corporation, Kobe, Japan) surgical robotic system was approved for use in Japan by the Japanese authorities in 2020. The Hinotori has a four-arm operational unit that adds an eighth DoF over the da Vinci X/Xi systems, potentially enabling smoother movements [19]. The surgeon console is a semi-closed design and allows the surgeons to manipulate the instruments using loop-like controls. A first-in-human trial of RARP in 30 patients was recently successfully undertaken following pre-clinical trials [19]. The authors suggested an equivalent performance to the da Vinci system, but further studies will be required to validate these findings. The relative similarity between the Hinotori and da Vinci systems does suggest that transitioning between systems may be easier than with other robotic systems. 

## 8. Revo-I

The Revo-I (Meere Company Inc., Yongin, Republic of Korea) has regulatory approval in South Korea granted by the Korean Ministry of Food and Drug Safety in 2017. The Revo-I has a closed surgeon console, four-arm bedside cart and vision cart [20]. The 7.4 mm instruments permit seven DoF and are reusable after sterilisation up to twenty times [20]. The first clinical study of Revo-I was for 17 patients undergoing Retzius-sparing RARP with successful completion of surgery and no conversions to open or laparoscopic surgery [20]. Subsequent comparisons have been made between the Revo-I and Da Vinci systems, although notably with the previous generation Si model, where there was no difference in short-term oncological times [39]. The authors also reported that the da Vinci Si system did have shorter operative duration but also a longer length of stay compared to the Revo-I [39].

## 9. Hugo

The Hugo RAS system (Medtronic, Minneapolis, MN, USA) was granted European approval for urological and gynaecological procedures in 2021, which broadened to include general surgery in 2022. It has an open surgeon console, four-individual arm carts and a systems tower [40]. Similar to the Senhance system, the 3D surgical display can be seen with dedicated glasses. An 11 mm port is used for the endoscopic camera with 8 mm ports for surgical instruments [40]. Initial reports of undertaking RARP with the Hugo system have recently been published with no intra-operative complications or technical failures identified [41,42]. However, these case series are of fewer than ten patients each and larger, multi-centre series are anticipated in the near future.

## 10. Avatera

**Figure 3:**
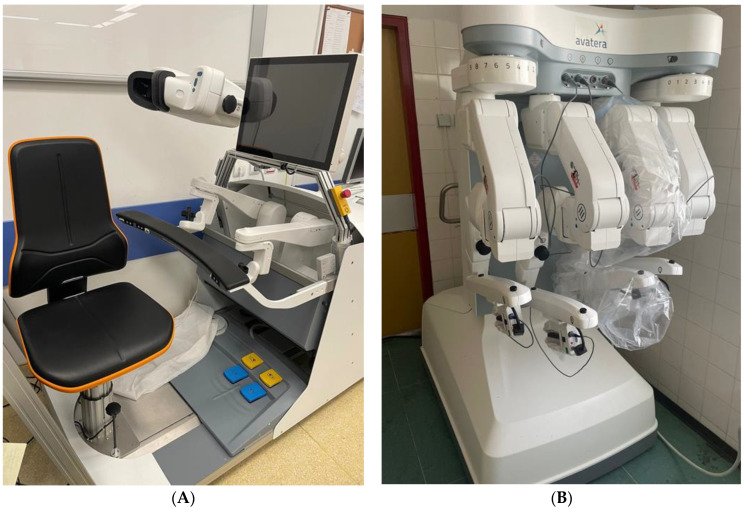
Avatera robotic system. (**A**) Closed surgeon’s console. (**B**) Robotic cart with four arms in the stored position.

The Avatera RAS system (Avateramedical, Jena, Germany) was awarded European approval in November 2019, primarily for use in urology and gynaecology [43] (Figure 3). It features a closed surgeon console unit and a robotic cart with four arms that use disposable 5 mm diameter instruments [43]. Initial studies undertaking six RARC with intracorporeal urinary diversion and six radical nephrectomies, respectively, in anaesthetised live porcine models reported no mechanical issues or major complications encountered [44,45].

## 11. Future Developments

The Dexter RAS system (Distalmotion, Épalinges, Switzerland) has European CE approval and differs from alternative RAS systems by offering ‘on-demand robotics’ at a lower cost [46]. The system is intended to allow an easy transition between laparoscopy and robotic access during an operation with two robotic arms that can be free-standing or fixed to the operating table with a sterile robotic console [46]. The company has recently announced completion of RARP and Millin’s prostatectomy using the Dexter system, but no formal evaluation has yet been published [47]. Johnson & Johnson’s Ottava RAS system is in development, a six-arm system that is designed to integrate into the operating table, thereby saving space and improving flexibility [48]. Whilst this may represent a significant development in the versatility of RAS systems, it is currently unclear when Ottava will be released.

Although RAS systems have been used in the management of renal tract calculi, these have predominately been utilised in select cases where conventional treatments have failed or have been deemed unsuitable [49]. However, the Monarch Platform robotic surgical system (Auris Health Inc., Redwood City, CA, USA) has recently been granted FDA approval for endourology use, having initially been approved for robot-assisted bronchoscopy [50]. Early findings in porcine models have recently been presented using the Monarch Platform in both percutaneous nephrolithotomy and ureteroscopy with results comparable to conventional devices [51].

Outcomes for RARP have been extensively investigated and tend to support improved early functional outcomes, but studies are less unanimous regarding longer term functional and oncological outcomes. Consequently, current EAU guidelines acknowledge that while RARP has become the preferred minimally invasive approach, it does not currently advocate any one approach (open, laparoscopic or robotic) over another [2]. 

A benefit of RASS is that the surgeon can operate on a patient away from the operating table. Typically, at a console within the same operating theatre, but there is the potential for robotic surgery to be combined with telemedicine to allow a surgeon to operate on a patient that is in a distant location and even in a different continent [52]. This could allow for improved accessibility to specialist healthcare with reduced need for travel, with particular benefits to those in rural areas or battlefield locations. Although the first transatlantic robotic surgery was first successfully performed 20 years ago, the use of remote robotic surgery is not widespread [53]. Current issues limiting its use are concerns with connectivity issues, time-lag and legal issues [52].

## 12. Barriers to Robot-Assisted Surgery

Several barriers exist to the uptake of RAS in both developed and less developed countries including cost, accessibility, training opportunities and perceived usefulness.

## 13. Accessibility

Whilst surgical robots are becoming ever more abundant, with 1347 da Vinci systems being shipped in 2021, these are predominately located in wealthy European, North American and Asian nations [16]. A 2020 study in England revealed that of the 149 acute NHS trusts in the country, 48 trusts had a total of 61 robots, with just over 10,000 procedures performed per annum [21]. The vast majority of these were urological procedures (84%); however, the study does highlight that even in a relatively small country such as England there is disparity in accessibility of patients to robotic surgical centres. Many areas of the country, typically more rural locations, are prohibitively far away from a robotic centre to benefit from it [21]. This finding is likely to be more pronounced in less developed countries and even in large, developed countries that have low population densities.

## 14. Cost

Cost is a major barrier, with both the initial cost associated with acquisition and the costs associated with maintenance and consumables. The da Vinci Xi retails at around 1.75 million USD, although this is variable by region and even hospitals, depending on individual contracts [21,54]. To obtain a benefit, centres need to be using the robotic system regularly to reduce costs, and this requires several surgeons, often across many specialities, to be sufficiently trained in the use of the robot. An Australian study calculated that median cost associated for the use of RAS alone, without additional ward and critical care costs, was AUD 8828 (USD ~ 5980) across several specialities including predominately urology [54]. These costs accounted for implementation costs (RAS acquisition and theatre modification), maintenance and consumables, with the cost per procedure being volume dependent—an increased volume of surgery reduced the impact of the initial implementation costs.

A UK study also demonstrated this relationship as they found a 5.5-fold increase in the median cost per procedure for a centre performing 53 cases per annum compared to one performing 446 (GBP 8679 vs. GBP 1587) [21]. This supports the centralisation of robotic surgery services to high volume centres for economic reasons as well as the associated improvement in oncological outcomes also seen in high volume centres. The National Institute for Health and Care Excellence (NICE) in the UK recommends that centres purchasing a RAS system for RARP should be expecting to perform >150 cases per annum to ensure its cost effectiveness [55]. The European Association of Urologists (EAU) recognises that higher volume centres also have improved oncological outcomes, but it does not set a minimum recommended number, citing a lack of evidence [2]. Ultimately, many centres, particularly those in less economically developed countries, will simply be unable to afford the prohibitively expensive acquisitional and maintenance costs associated with RAS at the current market rates. Leasing a robotic surgical system is an alternative to purchasing a robot outright, thereby avoiding the initial outlay, with 23% of UK centres providing robotic surgery in this way [21]. Intuitive Surgical reported an increasing trend for lease or usage-based schemes with them accounting for 38% of da Vinci 2021 sales compared to 33% in 2020 [16].

## 15. Cost Comparison

Several groups have conducted cost analyses between robotic, laparoscopic and open surgery approaches, with radical prostatectomy commonly being researched. A comprehensive cost-analysis considers not only the costs directly associated with the procedure but also the wider economic costs from social care and the impact from loss of earnings and associated taxation. A Swedish multi-centre trial of 2638 men comparing RARP to RRP estimated an increased cost of USD 3837 (USD 2747–4928) per robotic procedure with a two-year follow-up, and found that this was sensitive to the volume of RAS procedures undertaken [56]. Bijlani et al. in 2016 suggested that robotic surgery was economically favourable compared to RRP in the US when economic modelling was conducted for estimated costs to include management of complications [57]. The expenditures associated with the increased length of stay associated with RRP are a substantial contributor to the cost [57]. A systematic review investigating cost-effectiveness of RARP versus open and laparoscopic prostatectomy found RARP to be more costly but provided greater quality adjusted life years (QALY) and greater societal economic potential through more years spent working and associated taxation contributions, but the conclusion is somewhat limited by the heterogeneity of the studies included [58]. Interestingly, a recent cost-comparison of RARC in European countries identified that just 16% of the total cost was attributable to the robotic system and instruments with the operative duration and length of hospital stay being greater determinants of cost [59].

There are inevitably large discrepancies in the cost analyses due to the immeasurable number of variables that can be included. However, having high-volume centres where the RAS system is regularly used will reduce the cost-per-procedure, thereby improving economic viability.

## 16. Training and Learning Curves

During the infancy of robotic surgery in urological surgery, most of the early adopters transitioned from a different surgical approach (open and/or laparoscopic). Consequently, it was the existing senior urologists, who had previously completed their training, that were having to learn how to utilise RAS at a later stage in their career, thus lessening opportunities for trainees to begin developing these skills in the earlier stages of their careers [60]. Fortunately, robotic surgery lends itself to simulation-based training to develop skills and competence prior to operating, and structured training programmes are being developed [60,61]. However, there is some evidence to suggest that trainees are able to acquire competence in robotic surgery at an earlier stage, possibly as less interference from having previously learnt an alternative surgical approach and having to adjust from this [62].

The learning curve is a crucial concept in RAS and is characterised by the period in which surgeons initially develop an improvement in performance over time followed by a plateau phase in which there is limited further improvement [22]. Learning curves typically use functional outcomes such as operative time, blood loss, lymph node yield or length of stay as surrogates for technical performance [22]. A 2022 systematic review of the learning curve for robot-assisted radical cystectomy (RARC) demonstrated a wide-range of 10–50 procedures being required for trainees to reach the plateau phase for RARC when considering operative time and lymph node yield [63]. The wide range is similarly found in other RAS procedures and it limits the benefits of assessing learning curves and using this information to inform training programmes due to great inter-trainee variability [22,63]. An additional challenge of RAS is that each system itself differs and there is a learning curve when transitioning between systems, even for experienced surgeons, and this may present increasing challenges as a wider array of systems are made available.

## 17. Limitations of Robotic Surgery

Adopting RAS at an institution not only necessitates high initial expenditure and appropriately trained surgeons, but the whole surgical team must be adequately trained, including the scrub team and first assistant. There are benefits in investing the resources however, as a cohesive, well-trained team can efficiently complete the setting up and docking of the robot to the patient, whilst having an experienced assistant surgeon at the bedside can be associated with reduced operative duration [64,65].

Mechanical failure can occur with RASS, and this has been reported as resulting in patient harm. In a study of 14,141 patients, 16 patients were harmed as a result of RASS malfunction, 13 of which were mild and self-resolved, whilst the others resulted in external iliac vein, urethral and ileal injury, respectively [66]. A study of 10,267 consecutive cases in South Korea using the da Vinci RASS reported a mechanical failure rate of 1.8% (185/10,267) across a range of urological and non-urological procedures [67]. The most common reason was instrument failure in 70.3%, which was resolved by exchanging for a new instrument. Mechanical failure or malfunction (17.3%) and system error (12.4%) accounted for the remaining cases, with seven needing conversions to open or laparoscopic surgery. There were no deaths associated with mechanical failure of the robotic system in the series and the authors reported lower rates of mechanical failure as both the da Vinci system improved and the surgical expertise increased over the eight-year study period [67].

RAS requires specific patient positioning depending on the procedure being performed and this can be associated with peripheral nerve injuries [68]. The steep Trendelenburg position often adopted in RARP can be associated with ophthalmic (corneal abrasion and visual loss), nerve (peripheral nerve palsy) or musculoskeletal injuries (compartment syndrome or rhabdomyolosis) [69]. In a study of more than 175,000 patients undergoing radical prostatectomy, an ophthalmic- or nerve-related complication relating to patient positioning in robotic surgery occurred in 0.17% and 0.16% of cases, respectively, with no significant difference to open surgery when adjusted for patient- and hospital-related factors [69]. Consensus guidelines recommend limiting the steep Trendelenburg position (≥30°) whenever possible and only for the shortest time necessary where it is required; additionally, it should be avoided altogether in high-risk patients as it can reduce cardiac output [70]. An extra-peritoneal approach can be used where possible to reduce the angle of Trendelenburg and the CO_2_ insufflation pressure required [70].

The environmental impact of healthcare and its role in climate change is becoming an increasingly important topic, and surgery is a large contributor to greenhouse gas emissions through the production and disposal of single-use instruments, anaesthetic gas agents and energy use [71]. These can be substantial, and a recent systematic review estimated that the greenhouse gas production was 43.5% higher for robotic surgery compared to the equivalent laparoscopic procedure [72]. A single RAS case can result in emissions equivalent to 814 kg of carbon dioxide, which is the same quantity of emissions produced by a petrol-powered car driving 3658 km [72]. While RASS were safely used during the COVID-19 pandemic, it was employed only where the resources permitted its use [73].

## 18. Conclusions

Robotic surgical systems undoubtedly have benefits from both the surgeon and patient perspective, particularly with regards to urological oncology surgery. The market dominance of the da Vinci system over the last two decades has seen RARP as the preferred approach for prostate cancer surgery for many surgeons, with surgical technique evolving over this timeframe. Cost remains a major barrier to increasing uptake but the increasing competition in the market and associated technological advancements brings a promising future for a new era of robotic surgery in urology.
